# Interoceptive awareness and self-regulation contribute to psychosomatic competence as measured by a new inventory

**DOI:** 10.1007/s00508-020-01670-5

**Published:** 2020-05-19

**Authors:** Christian Fazekas, Alexander Avian, Rita Noehrer, Franziska Matzer, Christian Vajda, Hans Hannich, Aljoscha Neubauer

**Affiliations:** 1 Department of Medical Psychology and Psychotherapy, Medical University of Graz, Auenbruggerplatz 3, 8036 Graz, Austria; 2 Institute for Medical Informatics, Statistics and Documentation, Medical University of Graz, Graz, Austria; 3 Institute of Medical Psychology, University Medicine Greifswald, Greifswald, Germany; 4 Department of Differential Psychology, Institute of Psychology, Karl-Franzens University Graz, Graz, Austria

**Keywords:** Psychosomatic medicine, Psychophysiology, Interoception, Self-control, Questionnaire design

## Abstract

**Background:**

The interrelation of interoception, cognitive appraisal of bodily signals and conscious self-regulatory behavior is insufficiently understood although it may be relevant for health and disease. Therefore, it was intended to develop a novel self-report measure targeting this link.

**Methods:**

Item development was theoretically based on the multidimensional conceptual framework of the psychosomatic intelligence hypothesis and included an iterative process of refinement of items. In a preliminary test a principal components analysis (PROMAX rotation) and item analysis were calculated for item reduction. In the field test an item response theory approach was used for development of final scales and items. For validation purposes, associations with established measures of related constructs were analyzed.

**Results:**

The final 44-item questionnaire consisted of 6 interrelated scales: (1) interoceptive awareness, (2) mentalization, (3) body-related cognitive congruence, (4) body-related health literacy, (5) general self-regulation, and (6) stress experience and stress regulation. Psychometric properties of this instrument demonstrated good model fit, internal consistency and construct validity. According to the validation, the final instrument measures a form of competence rather than intelligence and was termed the psychosomatic competence inventory.

**Conclusion:**

Interoceptive awareness and conscious body-related self-regulation seem to jointly contribute to a basic competence which may serve homeostatic/allostatic control; however, further research is needed to confirm the reported preliminary findings in a large-scale test.

## Introduction

The detection and awareness of one’s bodily state plays a fundamental role in human physiology and human behavior [1–3]. This perception of bodily sensations is generally termed interoception. In its broad sense, interoception does not only refer to perceiving signals from the viscera as it was originally defined, but to all perceptions of bodily signals and bodily states, regardless of what information the brain uses or generates to construct this subjective experience [1]. The continuing bodily experience occurs as an iterative process from moment to moment, and consciously from time to time. Throughout this article, this inclusive meaning of interoception is referred to.

For a vast range of biological, psychological and social aspects of human life, interoception is closely linked to both conscious and non-conscious self-regulation and to health-related behavior. This link enables regulation of basic physiological needs, for example through water and food intake or through regulation of bodily activity, e.g. for reasons of subjective well-being [4, 5]. In addition, it may positively influence mental health [6] but can also lead to feelings of threat and discomfort and aggravate symptom burden, e.g. in patients with panic disorder [7]. Furthermore, interoceptive signals may influence the doctor-patient relationship in daily practice by contributing to transference and bodily countertransference [8, 9].

Interoception can be accompanied by cognitive appraisal, which raises the widely unexplored issue of a potential top-down impact of higher cognitive function on the link between interoception and self-regulation. According to the psychosomatic intelligence hypothesis (PI-hypothesis) [10, 11], the level of cognitive ability can modulate interoceptive awareness, mentalization of interoceptive signals, interoceptive learning, interoceptive memory, conscious interoceptive predictions, body-related decision making, and conscious self-regulation based on somatic information. In this concept, it is posited that cognitive ability can facilitate a differentiated, rapid and efficient processing of somatic information. Concerning the aspect of rapid and efficient processing, the PI-hypothesis bears analogy to the neural efficiency hypothesis of intelligence [12]. Several studies reported a positive correlation between IQ and speed of perception [13, 14]. Regarding its core content of differentiated processing of somatic information, the PI-hypothesis assumes an impact of earlier experiences with somatic signals and related knowledge about oneself, and an impact of prior experiences with efforts regarding body-related self-regulation on current self-regulatory behavior. It also posits individual variability in awareness of environmental influences on somatic information. Thus, the PI-hypothesis is based on the following five distinctive body-related dimensions: (1) interoceptive awareness, (2) mentalization, (3) analysis of incongruence, (4) knowledge, and (5) self-regulation. The third dimension, analysis of incongruence, is assumed to foster rapid awareness of incongruence/congruence concerning the intraindividual situation of the “material me” as well as concerning the individual environment transactions with impact on the “material me”. In the PI-hypothesis it is stated that any subdomain of intelligence needs to be significantly correlated with general mental ability (g) as a core information processing ability in order to justify the term intelligence [11]. Otherwise, the term psychosomatic competence should be applied in order to describe self-regulatory competence in dealing with somatic information.

In summary, the PI-hypothesis refers to cognitive aspects and skills in phases of body-related self-regulation and decision making. It suggests that an individual’s general mental ability may be involved in cognitive processing of interoceptive signals. Therefore, it is assumed that individuals with higher psychosomatic intelligence tend to be more successful in detecting, interpreting and managing interoceptive signals and transforming these signals into personally adequate internal or external behavior and behavioral patterns. For example, higher psychosomatic intelligence may go along with earlier detection of lacking physical activity for physical well-being and finding ways to integrate bodily activity in everyday life more easily. Generally, it may contribute to better regulate and respond to physical needs, e.g. concerning recovery, to overcome hindrances regarding personal health goals, e.g. for healthy nutrition, and to behave socially and emotionally competent without suffering physically in an unnecessary way, e.g. by constantly suppressing relevant somatic information leading to increased allostatic load. As psychosomatic intelligence is assumed to result in more adequate options for bodily self-regulation it may have provided an evolutionary advantage by integrating awareness of interoceptive signals in social behavior and by integrating interoceptive signals in decision making regarding consciously initiating challenging bodily activities, e.g. in hunting.

A novel conceptualization for a body-related cognitive ability or competence seems justified from a neuroscientific and a health scientific perspective. Perception of bodily sensations is assumed to be influenced by predictive coding which may confuse cognitive appraisal. The embodied predictive interoception coding (EPIC) model [15] suggests that the brain actively generates interoceptive predictions based on prior interoceptive sensations that interfere with ascending interoceptive signals for reducing predictive error. The concept of predictive coding is increasingly considered to influence vulnerability for mental and physical illness [16, 17]. The EPIC model posits that homeostatic-allostatic control and interoception are unified within an integral neural architecture [15]. Recent neurobiological evidence seems to support this notion of a unified allostatic/interoceptive brain system as a core function of the central nervous system [18]. Large-scale brain systems seem to integrate allostatic and interoceptive information to regulate the internal milieu (allostasis) based on representations of the internal milieu (interoception). These systems seem to encompass the default mode and salience networks which serve as a high-capacity backbone for integrating information across the brain (including cognition, attention, emotion, perception, stress, and action) [18–20]. Interestingly, in line with the view of the central nervous system (CNS) as a predictive coding machine, recent evidence suggests with respect to functional somatic syndromes that, in addition to sensory input and bottom-up processes, top-down predictions, such as expectations, substantially influence interoceptive perception and chronic bodily distress [21, 22]. From a health science perspective, Contrada and Coups pointed to similarities between models of health-related self-regulation and certain definitions of intelligence in 2003 [23] by referring to the triarchic model of Sternberg [24]. Consequently, these authors argued for the existence of forms of somatic intelligence and suggested implications for processes of self-regulation involving the detection and interpretation of bodily sensations. Yet, research on health and illness cognition has not been generally framed in terms of intelligence or ability constructs [23], although general mental ability (g) has been shown to be substantially related to auditory and visual discrimination [13].

Despite rapidly increasing interest in studying interoception and its role in conscious self-regulation there is a scarcity of self-report instruments targeting this research field as most questionnaires on interoceptive awareness were based on the earlier conceptualization of body awareness as proxy measures for anxiety [25]. A more comprehensive and refined approach to the measurement of interoceptive sensibility, including positive interoceptive phenomenology associated with meditative experience, is the multidimensional assessment of interoceptive awareness (MAIA) [26]. Nevertheless, to our knowledge, none of the existing self-report instruments including the MAIA taps on the proposed link between cognitive appraisal of interoceptive information, domains of cognitive ability, e.g. interoceptive learning and memory, and active self-regulation. Therefore, this study aimed to explore if a brief and sufficiently valid and reliable self-report tool can be developed based on the PI-hypothesis in order to measure interoceptive awareness, cognitive appraisal and conscious self-regulation as supposedly interrelated domains. It was predefined that this tool can only be assumed to represent the construct psychosomatic intelligence if validation demonstrates a substantial correlation with other measures of intelligence. Otherwise this tool should be considered to measure the construct psychosomatic competence which comprises the same set of body-related abilities and skills as psychosomatic intelligence but is unrelated to performance tests for intelligence.

## Methods

### Item development

An expert panel consisting of three psychologists and two MDs conducted the initial questionnaire development in German language within a series of 18 working group meetings. In addition, 25 colleagues and students of 2 departments of 2 Universities were repeatedly involved in this process. The working group of panel members operationalized psychosomatic intelligence based on the five body-related dimensions (1) interoceptive awareness, (2) mentalization, (3) analysis of incongruence, (4) knowledge, and (5) self-regulation. These five dimensions were regarded as interrelated key concepts. Interoceptive awareness was assumed to be particularly activated under certain circumstances, e.g. in stressful and in social situations, in decision making, and in changing behavior patterns towards health-related behavior [27]. Definitions of all other dimensions also referred to bodily signals and therefore differed from related theoretical concepts and their operationalization in questionnaires, e.g. self-regulation as defined by Schwarzer focuses on regulation of attention and emotion in pursuing goals [28]. Prototype items were defined for all five dimensions. Then a list of variables was developed for all five dimensions. Bodily signals that typically and frequently lead to cognitive appraisal and conscious self-regulation were collected as variables and stimuli for item development. Self-regulatory activities encompassed both specifically body-related self-regulation and general self-regulatory activity, like in general decision making. In a next step, more than 25 items were generated for each dimension. Then the panel decided on 20 items for every dimension by eliminating items with similar content. A random sequence of these preliminary 100 items was determined, a 6-point Likert type response scale was chosen as answering format (strongly disagree, disagree, somewhat disagree, somewhat agree, agree and strongly agree), and the instruction was added. These items were iteratively presented to the group of colleagues and students and revised by the panel concerning understandability and precise and clear wording. Thus, 13 items were modified. Afterwards, persons in the social environment of panel members were asked to answer the current version of the questionnaire and to report any difficulties in understanding and responding, which led to minor changes in the instruction and the format of the questionnaire.

After this initial phase of item generation ethics committee approval was obtained from the ethics committee of the Medical University of Graz for the further stepwise development of the questionnaire which included a cognitive survey of the preliminary version, a pre-test and a field test. All methods in the following multistep approach were performed in accordance with the relevant guidelines and regulations. Informed consent was provided by all study participants. The cognitive survey used a think aloud approach and allowed estimating if the wording of items would lead to inconsistent interpretations by different individuals. A convenience sample of 9 persons (7 female) with an age range between 21 and 67 years, different employment status and educational background participated in this survey. They completed the 100-item questionnaire while speaking out loud their corresponding thoughts and associations. Transcripts of participants’ cognitions were discussed in an expert panel (one MD, two psychologists) and all items were re-evaluated. In accordance with the main suggestions of the cognitive survey some similar items were deleted and some items with ambiguous wording were clarified by exemplification. As a result of the cognitive survey, eight items needed to be deleted. The panel decided to drop another two items due to similarity resulting in a 90-item version of the questionnaire with all 5 dimensions being represented by 18 items each.

### Pre-test

The pre-test was based on the 90-item version of the questionnaire and aimed at an empirical exploration of the factor structure and a first item analysis. Study participants in the pre-test were primarily recruited at the 2 involved universities and consisted of 177 persons (115 females, 62 males; 80% students) between 15 and 69 years with a mean age of 27 years (SD = 9.7 years).

#### Empirical exploration of factor structure and item analysis

A principal components analysis (PROMAX rotation) was calculated, as such oblique rotation can be used if correlated factors are expected. As compared to an, also computed, orthogonal rotation (VARIMAX) a similar factor structure was found, thus favoring PROMAX rotation due to the hypothesized correlations of components. To determine the number of factors to retain, Horn’s parallel analysis and the criterion of eigenvalue >1 were applied. Items with more than 5% missing values or a difficulty *p* < 0.1 or *p* > 0.9 or an item discrimination (correlation of the item with the test score) of r_it_ < 0.2 were excluded.

### Field test

The field test was conducted with the aim of determining the final factor structure in a heterogeneous sample and a first validation of the final German version of the questionnaire. It also aimed at further item reduction of the version of the questionnaire, which had resulted from the pre-test in order to create a more economic instrument with a minimum of handling time.

#### Study participants

As compared to participants in the pre-test, respondents in the second sample should be more representative for the population. Therefore, a heterogeneous group was included. Study participants within a predefined age range were intended to represent groups with different educational background. Besides sufficient knowledge of the German language no further inclusion or exclusion criteria were applied. Participants were recruited among employees of a local industrial goods production company and employees of the local university and hospital, among students and among other persons who were interested to participate due to public announcement of the study. Characteristics of study participants are given in Table 1.

**Table 1:** Sample characteristics (*n* = 103)

		Mean ± SD (range) or *n* (%)
Age (years)		40.5 ± 14.4 (range: 18–75)
Sex	Male	42 (40.8%)
	Female	61 (59.2%)
Body mass index		23.9 ± 3.5 (range: 17.3–33.3)
Smoking status	Smoker	19 (19.4%)
	Nonsmoker	84 (81.6%)
Marital status	Unmarried	49 (47.6%)
	Married	48 (46.6%)
	Divorced	5 (4.9%)
	Widowed	1 (1.0%)
Living situation	Living alone	16 (15.5%)
	Living with partner and/or children	68 (66.0%)
	Living in a shared apartment	6 (5.8%)
	Living with parents or relatives	13 (12.6%)
Highest level of education	Secondary education first stage/second step of basic education	38 (36.9%)
	Upper secondary education, which provides direct access to tertiary education	26 (25.2%)
	Tertiary education	39 (37.9%)
Employment status	Employed	83 (80.6%)
	Unemployed	20 (19.4%)

#### Statistical analysis

In the field test items were analyzed using an item response theory (IRT) approach. A generalized partial credit model was applied to model the responses (estimation method: quasi-Monte Carlo method). For this analysis, the R‑package mirt (version 1.25) was used [29, 30]. For the explorative factor analysis different numbers of factors were considered. Since within the mirt package only one number of factors can be analyzed at once, this analysis was done for several numbers of factors separately. Therefore, a one factor model, a two factor model a.s.o up to a nine factor model were analyzed. Results of these different models were compared afterwards to come up with the best fitting model. Beginning with the one-factor solution each solution was compared to the next higher solution using likelihood ratio tests until the more complex model did not result in a significant better model fit. Starting with this best model, the number of items showing factor loadings >0.4 per each factor was analyzed. The five items with factor loadings >0.4 within one factor were considered adequate. If within the best fitting model factors did not have an adequate number of items with sufficing factor loadings the next less complex model was analyzed. In the final model, items with double loadings or highest loadings ≤0.3 were excluded. Model parameters and final factor loadings were estimated using the remaining items. Model-to-item fit (S-χ2 statistic) and overall model fit (root mean square error of approximation, RMSEA; Tucker-Lewis index, TLI; comparative fit index, CFI) were also examined. A better model fit is associated with lower RMSEA and higher TLI and CFI values. Since analyses in smaller sample sizes tend to result more often in an increased type II error [31] the following cut-off scores were chosen to indicate an acceptable model fit: RMSEA ≤0.1, TLI ≥0.9, and CFI ≥0.9. Marginal reliability (ρ) was calculated as it is an estimate of the overall reliability of a factor based on the average conditional standard errors.

### Validity

For a first estimate of construct validity, the correlations with other measures were analyzed using Pearson’s correlation coefficient or Spearman’s rank correlation coefficient, as appropriate. A correlation coefficient r > 0.3 was considered relevant. Furthermore, differences between defined groups (e.g. sex, marital status) were analyzed using t‑tests or ANOVA (post hoc analysis with Bonferroni correction). For significant differences Cohen’s d with 95% confidence intervals were calculated. Data from the field test were used for these analyses. Regarding construct validity, the dimension (1) interoceptive awareness of the PI-hypothesis was assumed to be correlated with self-consciousness. The dimensions (2) mentalization, (3) analysis of incongruence, and (4) knowledge were assumed to show correlations with performance tests for intelligence. Furthermore, the dimension (5) self-regulation was expected to correlate with another self-regulation scale and with self-efficacy. With respect to criterion validity, correlations with the level of physical activity and the number of health complaints were expected.

Following questionnaires and tests were used for validity evaluations: self-consciousness was studied with the German modified version of the self-consciousness scale which separately measures self-monitoring disposition in the public and private domain [32–34]. General intelligence was assessed by Raven’s advanced progressive matrices (APM) [35]. Attention was examined with the d2 attention test [36, 37]. Self-regulation was measured by the German version of the self-regulation scale [28]. Self-efficacy was studied using the German version of the generalized self-efficacy scale [38]. The German version of the international physical activity questionnaire (IPAQ) was applied to assess physical activity over the last 7‑day period [39, 40]. The number of currently perceived bodily complaints, such as headaches, palpitations, diminished appetite and fatigue, was investigated by the German bodily complaints list [41].

## Results

### Pre-test

A high number of factors (*n* = 23) had eigenvalues >1. Using Horn’s parallel analysis resulted in a reduction of this high number of factors to five factors. After eliminating all items with double loadings (items loading on two factors within a scope of 0.1) and highest factor loadings ≤0.3 and low item discrimination (r_it_ < 0.2), a version of the questionnaire with 65 items remained. Prior to deciding on removing items, a content evaluation was performed with respect to relevance and content coverage of those 25 items which were finally excluded. No item had to be excluded because of too high (*p* < 0.1) or too low (*p* > 0.9) item difficulty. Each item was assigned to one of five factors. The first factor included 15 items, the second factor 14 items and factors 3, 4, and 5 12 items each. Factors 1 and 2 could explain 9% of variance each, factors 3 and 4 could explain 8.6% of variance and factor 5 explained 7.2% of variance.

### Field test

A model fit was analyzed for the responses of 103 respondents (female: 59.2%; age: 40.5 ± 14.4 years) to the 65 remaining items of the pre-test. Up to the eight-dimensional model, the model with more dimensions fitted data significantly better than the less-dimensional model (9 vs. 8 ∆χ^2^ df = 57 = 30.4, *p* = 0.999, 8 vs. 7 ∆χ^2^ df = 58 = 109.2, *p* < 0.001, 7 vs. 6 ∆χ^2^ df = 59 = 118.7, *p* < 0.001, 6 vs. 5 dimensions: ∆χ^2^ df = 60 = 103.7, *p* < 0.001; 5 vs. 4 dimensions: ∆χ^2^ df = 61 = 150.5, *p* < 0.001). Factor loadings of the different models showed that within the eight-dimensional model six dimensions and within the seven-dimensional model four dimensions and within the six-dimensional model one dimension had fewer than five items with adequate factor loadings. Since the five-dimensional model also had one dimension with less than five items with sufficing factor loadings (>0.4), the six-dimensional model was chosen. Factors 1 and 2 could explain 8% of variance each, factors 3, 4 and 5 could explain 7% and factor 6 explained 3% variance. The overall model fit of the six-dimensional model was good, as indicated by the CFI and TLI (CFI: 0.917, TLI: 0.912) and acceptable as indicated by the RMSEA (0.088, 90% confidence interval, CI: 0.080–0.095).

#### Final scales and items

According to the content of the six factors they were named (1) stress experience and stress regulation, SER, (2) body-related health literacy, BHL, (3) body-related cognitive congruence, BCC, (4) mentalization, M, (5) interoceptive awareness, IA, and (6) general self-regulation, GSR.

The final instrument thus comprised six scales with five to nine items per scale and consisted of a total of 44 items. Since no significant correlation with the applied intelligence test was found as described below (Table 5), this instrument was named “psychosomatic competence inventory” (PSCI). Definition of final scales and corresponding items are given in Table 2. Translation to English was conducted based on the final German version by two English native speakers independently translating forth and back.

**Table 2:** Final scales and items of PSCI

Scales	No	Items
Stress experience and stress regulation (SER)	SER‑1	When I recognize that I have less energy than expected, I know why this is so
	SER‑2	When I am dissatisfied with my life situation, I know the cause
	SER‑3	When I suddenly feel physically weak, I know why
	SER‑4	I can get back into my stride, if things are not going so well (e.g. professionally, personally, health-wise …)
	SER‑5	I can differentiate clearly between states of tension in individual muscle groups (e.g. my shoulders as compared to my arms)
	SER‑6	I can get myself going again without stimulants (like coffee, for example) even when I am tired
	SER‑7	I am capable to adjust readily to difficult professional and personal circumstances
	SER‑8	I know exactly how much stress I can take without overtaxing myself
Body-related health literacy (BHL)	BHL‑1	I know exactly how much exercise I need in order to feel physically well
	BHL‑2	I realize when I am lacking physical activity
	BHL‑3	I find it easy to experience my body in the present moment
	BHL‑4	I know exactly what I can do in order to feel physically well
	BHL‑5	I have sufficient theoretical knowledge on how to contribute to my own well-being
	BHL‑6	In certain situations (e.g. meetings, driving) in which I cannot satisfy my physical needs (e.g. relaxation, exercise), I have a method to regulate myself
	BHL‑7	I know which types of food I can tolerate well and which I cannot
	BHL‑8	I notice how different foods influence my physical state (positively or negatively)
Body-related cognitive congruence (BCC)	BCC‑1	I am capable to recognize the causes behind my physical sensations
	BCC‑2	Even when I am feeling good, I notice what my body is telling me
	BCC‑3	I know how to motivate myself with respect to my health goals
	BCC‑4	When I exert myself physically, I can readily estimate how much I can demand of myself
	BCC‑5	I can also apply and utilize my theoretical knowledge (e.g. about health, psychological crises) for myself
	BCC‑6	I examine my goals (professional, sporting …) to evaluate whether they are compatible with my health
	BCC‑7	I reach my goals, even if I must give up pleasurable things
	BCC‑8	Whenever I would like to change a situation (e.g. a lack of job satisfaction or well-being), several possibilities occur to me
	BCC‑9	When making important personal decisions (e.g. moving house), I consider their effects on my health
Mentalization (M)	M‑1	I can communicate my physical wellbeing precisely to others
	M‑2	I can describe my moods accurately
	M‑3	I know very well what my most important needs are (e.g. social contacts, closeness/distance, movement, change …)
	M‑4	I can describe my various physical conditions (fitness, well-being, energy level) well using language
	M‑5	When I relax, I can sense physical changes
Interoceptive awareness (IA)	IA‑1	I recognize when I lose my inner balance
	IA‑2	I sense when something is beginning to strain me, a conversation, for example
	IA‑3	I am aware how other people’s physical states (e.g. agitation, calmness, nervousness) affect me
	IA‑4	When I reach peak performance, I know it immediately
	IA‑5	In certain situations (e.g. with decisions) I consciously discern my intuition
	IA‑6	I notice different physical responses depending on the setting (e.g. pleasant or unpleasant social surroundings)
	IA‑7	I am consciously aware of sensations of physical pressure (e.g. an uncomfortable seat, a handshake)
	IA‑8	When I feel something is unpleasant (e.g. conversations, touching), I recognize the causes
	IA‑9	I sense many things intuitively
General self-regulation (GSR)	GSR‑1	At most I lose my composure only briefly, even when I cannot put an important plan into practice
	GSR‑2	Even when everything suddenly goes wrong, I remain capable of taking action
	GSR‑3	I do not allow myself to be diverted from my original goals, even when I slip back into negative habits
	GSR‑4	I register clearly when people are coming physically too close to me
	GSR‑5	I can judge in advance which situations (e.g. private, professional …) I should avoid

#### PSCI: factor loadings of items, factor correlations and marginal reliability

Factor loadings (Λ) of the final items of the PSCI are presented in Table 3. Marginal reliability for all six factors was acceptable to good (ρ = 0.79–0.90). As theoretically assumed, all factors were found to be correlated. Factor correlations and marginal reliabilities are given in Table 4.

**Table 3:** Factor loadings for items of PSCI

	Factor loading (Λ)					
No	Factor 1	Factor 2	Factor 3	Factor 4	Factor 5	Factor 6
SER‑1	0.822	–	–	–	–	–
SER‑2	0.783	–	–	–	–	–
SER‑3	0.823	–	–	–	–	–
SER‑4	0.535	–	–	–	–	–
SER‑5	0.555	–	–	–	–	–
SER‑6	0.686	–	–	–	–	–
SER‑7	0.586	–	–	–	–	–
SER‑8	0.553	–	–	–	–	–
BHL‑1	–	0.763	–	–	–	–
BHL‑2	–	0.538	–	–	–	–
BHL‑3	–	0.472	–	–	–	–
BHL‑4	–	0.878	–	–	–	–
BHL‑5	–	0.635	–	–	–	–
BHL‑6	–	0.777	–	–	–	–
BHL‑7	–	0.769	–	–	–	–
BHL‑8	–	0.424	–	–	–	–
BCC‑1	–	–	0.776	–	–	–
BCC‑2	–	–	0.476	–	–	–
BCC‑3	–	–	0.463	–	–	–
BCC‑4	–	–	0.685	–	–	–
BCC‑5	–	–	0.618	–	–	–
BCC‑6	–	–	0.615	–	–	–
BCC‑7	–	–	0.712	–	–	–
BCC‑8	–	–	0.232	–	–	–
BCC‑9	–	–	0.569	–	–	–
M‑1	–	–	–	0.873	–	–
M‑2	–	–	–	0.963	–	–
M‑3	–	–	–	0.591	–	–
M‑4	–	–	–	0.600	–	–
M‑5	–	–	–	0.704	–	–
IA‑1	–	–	–	–	0.467	–
IA‑2	–	–	–	–	0.523	–
IA‑3	–	–	–	–	0.689	–
IA‑4	–	–	–	–	0.539	–
IA‑5	–	–	–	–	0.423	–
IA‑6	–	–	–	–	0.681	–
IA‑7	–	–	–	–	0.523	–
IA‑8	–	–	–	–	0.619	–
IA‑9	–	–	–	–	0.533	–
GSR‑1	–	–	–	–	–	0.484
GSR‑2	–	–	–	–	–	0.639
GSR‑3	–	–	–	–	–	0.669
GSR‑4	–	–	–	–	–	0.416
GSR‑5	–	–	–	–	–	0.306

**Table 4:** Correlation matrix and marginal reliability of PSCI scales

	SER	BHL	BCC	M	IA	GSR
SER	*0.858*	0.683**	0.678**	0.618**	0.550**	0.683**
BHL	0.656**	*0.885*	0.629**	0.647**	0.553**	0.575**
BCC	0.665**	0.670**	*0.881*	0.659**	0.629**	0.632**
M	0.662**	0.695**	0.674**	*0.898*	0.706**	0.612**
IA	0.595**	0.658**	0.630**	0.629**	*0.876*	0.612**
GSR	0.517**	0.459**	0.575**	0.459**	0.519**	*0.791*

Values above the diagonal are correlations based on factor scores resulting from IRT estimations. Values below the diagonal are sum scores correlations. Diagonal values are marginal reliability values

*SER* stress experience and stress regulation, *BHL* body-related health literacy, *BCC* body-related cognitive congruence, *M* mentalization, *IA* interoceptive awareness, *GSR* general self-regulation

** *p* < 0.001

#### Characteristics of study participants and PSCI

Neither age nor BMI were found to correlate with the factors of the questionnaire. Furthermore, no difference in the factors of the questionnaire was found between groups according to sex, marital status, employment status, and smoking status. Overall significant differences were found in four PSCI factors for education (main effect: GSR *p* = 0.003; BHL *p* = 0.004, IA *p* < 0.001, M *p* = 0.001) and a tendency in the remaining two factors (SER *p* = 0.076; BCC *p* = 0.081). Respondents with tertiary education had higher IA (*p* < 0.001), M (*p* = 0.001), BHL (*p* = 0.012) and GSR (*p* = 0.003) values compared to respondents with lower secondary education. Respondents with tertiary education also reported higher M (*p* = 0.039) and BHL (*p* = 0.017) values compared to respondents with upper secondary education. Furthermore, respondents with upper secondary education reported higher IA values (*p* = 0.020) compared to respondents with lower secondary education. Concerning the living situation, a significant difference could be observed for SER (main effect *p* = 0.037). Post hoc analysis revealed that respondents living with partner and/or children had higher SER compared to respondents living with parents or relatives (*p* = 0.035). No other differences between groups could be observed.

### Validity

For a first evaluation of construct and criterion validity of the PSCI the correlations between its scales and related measures including instruments testing for self-consciousness, intelligence, attention, self-regulation, self-efficacy, physical activity, and bodily complaints were analyzed (Table 5).

**Table 5:** Correlation of the PSCI scales with validity measures

	IA	M	BCC	BHL	GSR	SER
Self-consciousness private	0.403**	0.231	0.371**	0.080	0.284*	−0.088
Self-consciousness public	0.339**	0.229	0.408**	0.121	0.149	0.052
Intelligence	0.242	0.042	0.088	0.023	0.205	0.027
Attention	0.335*	0.241	0.128	0.084	0.113	−0.031
Self-regulation	0.277*	0.283*	0.183	0.484**	0.513**	0.446**
Self-efficacy	0.307*	0.349**	0.419**	0.425**	0.568**	0.589**
Physical activity	−0.064	−0.073	0.076	0.128	0.086	0.021
Bodily complaints	−0.195	−0.200	−0.190	−0.287*	−0.372**	−0.312*

*SER* stress experience and stress regulation, *BHL* body-related health literacy, *BCC* body-related cognitive congruence, *M* mentalization, *IA* interoceptive awareness, *GSR* general self-regulation

*p < 0.05. **p < 0.01

The factors IA and BCC showed a positive correlation with private and public self-consciousness. In addition, GSR was positively correlated with private self-consciousness. None of the six factors showed a significant correlation with the intelligence test (r < 0.30), and only IA correlated positively with the performance in a concentration test (r = 0.335, *p* =0.012). All scales except BCC were positively correlated with self-regulation, and all scales showed significant correlations with self-efficacy.

No correlations with physical activity were found, whereas BHL, GSR and SER showed expected negative correlations with bodily complaints.

## Discussion

**Fig. 1:**
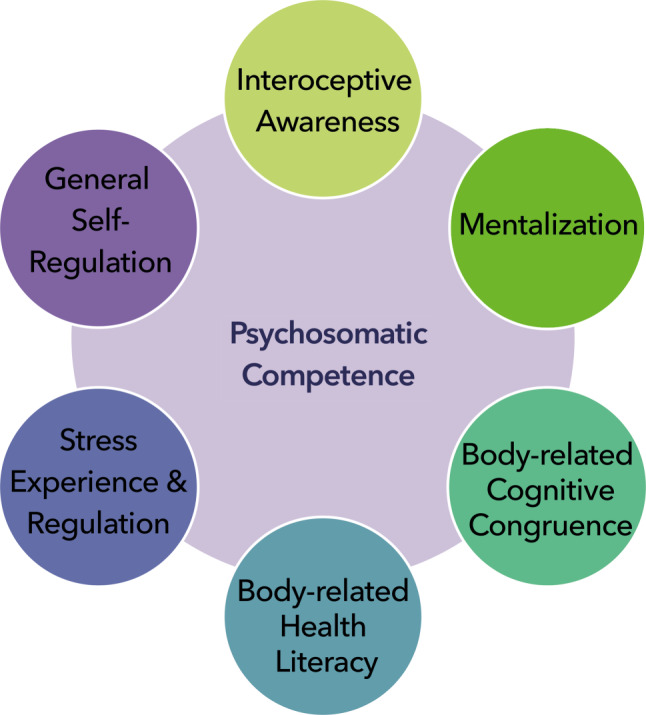
Psychosomatic competence refers to a basic human ability to consciously self-regulate bodily signals. The following correlated factors jointly contribute to self-reported psychosomatic competence: interoceptive awareness, mentalization, body-related cognitive congruence, body-related health literacy, stress experience and stress regulation, and general self-regulation

This study reports on the final version and psychometric properties of a new theory-based instrument on interoceptive awareness, cognitive appraisal and conscious self-regulation. The resulting final self-report measure was called PSCI. It consists of 44 items that are answered on a 6-point scale. These items relate to six factors named interoceptive awareness, mentalization, body-related cognitive congruence, body-related health literacy, general self-regulation, and stress experience and stress regulation which were found to be correlated in contributing to the measurement of psychosomatic competence (Fig. 1). Concerning the psychometric properties of the inventory, it demonstrated good model fit, internal consistency and construct validity.

**Fig. 2:**
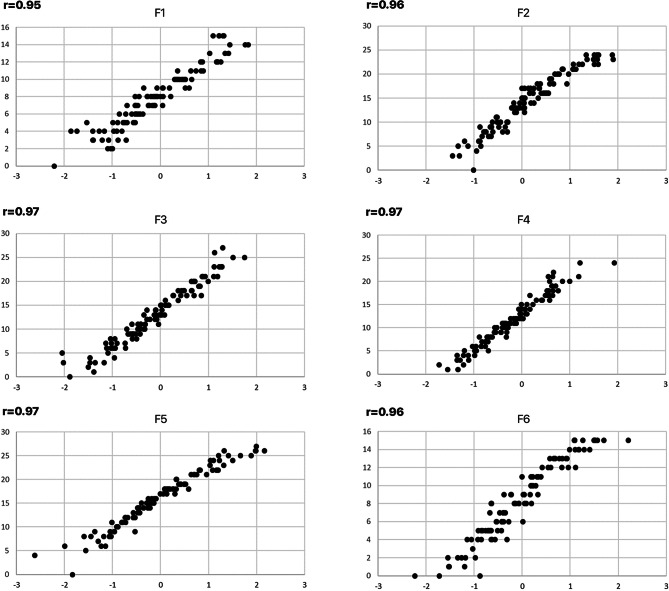
Correlation of the factor scores derived from the IRT model (X-axis) and sum scores (Y-axis)

Regarding the calculation of the PSCI, a precise computation of individual scores according to the applied IRT models requires an inclusion of the item intercepts and discrimination parameters of each item. While this procedure is acceptable for research projects or when using a digital device (e.g. answering the questionnaire using a tablet or smart phone), it is not practicable for daily routine work when using a paper and pencil test. Therefore, the use of a simple mean score for each factor instead of a factor score derived from an algorithm is preferable in such cases. Since these simple mean scores show high correlations (r > 0.95) with the factor scores calculated according to the IRT models (Fig. 2) the use of these mean scores is acceptable. A total sum for the PSCI can be easily calculated. Nevertheless, the use of exactly calculated scores should be considered whenever possible.

As the PI-hypothesis constituted the theoretical basis for the development of the PSCI, reported results should be discussed in relation to the underlying theoretical assumptions. A comparison of the final scales with the originally assumed dimensions is given in Table 6. It reveals both similarities and differences. Denomination of two scales of the PSCI, interoceptive awareness and mentalization, fully corresponds to these dimensions in the PI-hypothesis. Naming of two further PSCI scales, body-related cognitive congruence and body-related health literacy, slightly differs from the theoretical dimensions due to positive wording and specification. Furthermore, the dimension self-regulation of the PI-hypothesis is now represented by two PSCI scales, stress experience and stress regulation and general self-regulation (Table 6). In the scale stress experience and stress regulation items have clustered that address physically or mentally challenging situations, stress-related interoceptive sensations or stress regulation. This scale seems to correspond well with the specific role of interoceptive signals in self-regulatory stress management as described in the biopsychosocial model of stress [42]. Altogether, although the PSCI consists of six final scales as compared to five initial dimensions, in terms of content, PSCI scales seem to correspond well with the initially defined theoretical dimensions.

**Table 6:** Comparison of PSCI scales developed by using item response theory with theoretically developed dimensions of the PI-hypothesis

PSCI scales	Dimensions of PI-hypothesis
Interoceptive awareness (IA)	Interoceptive awareness
Mentalization (M)	Mentalization
Body-related cognitive congruence (BCC)	Analysis of incongruence
Body-related health literacy (BHL)	Knowledge
General self-regulation (GSR)	Self-regulation
Stress experience and stress regulation (SER)	

A substantial difference between the PSCI and the PI-hypothesis arises from the decision to describe this novel inventory as a measure for psychosomatic competence and not for psychosomatic intelligence. This decision was based on the correlations of PSCI scales with validity measures. While we found only one correlation between interoceptive awareness and attention and no correlation with a performance test on intelligence, all scales correlated positively with a measure of self-efficacy and, in addition, five of four scales correlated positively with a validity measure for self-regulation. Therefore, this new instrument was named PSCI according to the use of the term psychosomatic competence in the PI-hypothesis, which describes a self-regulatory competence in dealing with somatic information that is not associated with intelligence [11].

Interestingly, recent evidence from neuroscience also suggests that cognition and a unified allostatic/interoceptive brain system could concurrently provide a basic human regulatory competence to maintain balance of the internal milieu while signals from visceromotor cortices can immediately react to physical, social or mental challenges [15, 17, 18]. While autoregulation is generally viewed as a subconscious process, the factors of the PSCI may contribute to a basic cognitive competence which serves homeostatic/allostatic control. In combination with complementing methodological research approaches the PSCI could help explore this assumption; however, several limitations need to be considered regarding the development and final version of the PSCI. At first, it should be pointed out that no single self-report instrument, including the PSCI, can cover the range of interoceptive experience and related human behavior. In addition, for all self-report measures the problem of systematic errors according to the chosen way of data acquisition arises, e.g. due to social desirability. Given that up to now there is no external way of probing for psychosomatic competence a self-reporting tool is the only way to get this information.

A second limitation, which also applies to other questionnaires in the field of psychology, is the question of validity. Because of the lack of a gold standard, the validity question is never completely answered. Nevertheless, in a first effort to provide evidence of the questionnaire’s validity, some associations were found as expected (e.g. interoceptive awareness with attention), while other associations with validity measures were absent, thus contradicting our expectations (e.g. mentalization with intelligence and attention). Concerning criterion validity, as expected, the number of bodily complaints was related to some scales of the PSCI, yet, contrary to our expectations, physical activity, BMI and smoking did not show any noteworthy correlations. Therefore, especially measures of health behavior like physical activity, body mass index, smoking behavior or alcohol consumption should be included in future validation studies to prove or disprove if higher psychosomatic competence can predict better health behavior beyond established instruments and concepts. Additional studies with more participants are also needed in different populations, different settings and with different validity criteria to get a broader view on the construct validity and a perspective on the incremental validity of the questionnaire.

The generally high intercorrelations of the scales also need to be discussed as a potential limitation which suggests that these scales cannot be regarded as fully independent. Indeed, this assumption corresponds with the PI-hypothesis of a link between interoception, cognitive appraisal and conscious self-regulation as correlated, yet, distinctive dimensions.

Moreover, the small sample size resulted in cut-off criteria for fit indices that reduced the risk for type II error. Commonly used cut-off criteria for large samples sizes would have resulted in very high rejection rates (e.g. TLI ≥ 0.95; rejection rate for the true population model *n* ≤ 250: 28.9%) [29]. Nevertheless, the fit of the proposed model and therefore the proposed factor structure should be challenged in studies using bigger sample sizes. Furthermore, as more female respondents participated in the pre-test and field test a possible gender bias cannot be ruled out suggesting that further research in both sexes is needed. In addition, we did not observe a difference in the factors of the questionnaire between female and male respondents. Yet, recent findings suggest that females tend to notice bodily sensations more often and may better understand relations between bodily sensations and emotional states than men [43], whereas in another study higher levels of long-term self-regulation were reported by men as compared to women [44].

Finally, this study was conducted in healthy adults to probe for a basic human ability. Therefore, clinical validity and sensitivity of the PSCI still needs to be explored in clinical samples.

A major strength of the PSCI is its innovative approach to addressing interoceptive signals as health-related information implying an active self-regulatory competence in perceiving, interpreting and managing somatic information. The theory-driven development and good psychometric properties of this tool suggest that it could be relevant for neuroscientific, health-related and clinical research. The PSCI could thus contribute to better understanding and supporting of individual body-related self-regulatory skills associated with interoceptive sensations which are known to affect stress response, allostatic load, social behaviour, health and disease.
